# Editorial: Flavonoids, phenolics, and saponins in the diet for prevention and management of type 2 diabetes

**DOI:** 10.3389/fnut.2025.1611238

**Published:** 2025-06-09

**Authors:** Le-Le Yang, Na Xiao

**Affiliations:** ^1^Guangxi Key Laboratory of Special Biomedicine, School of Medicine, Guangxi University, Nanning, China; ^2^Zhuhai UM Science and Technology Research Institute, Zhuhai, China; ^3^College of Agronomy, Shandong Agriculture University, Tai'an, China

**Keywords:** type 2 diabetes mellitus, diet, flavonoid, mulberry leaf, Garcinia mangostana

Diabetes mellitus is expected to affect 642 million adults worldwide by 2040-the predominant form of which is type 2 diabetes mellitus (T2DM), with constantly rising in the global burden (1). The pathophysiology of T2DM is complex and involves multiple pathogenetic disturbances present in various organs and tissues, including the pancreatic β-cells of the islets, peripheral tissues, and digestive tracts, etc. (2). T2DM, characterized by persistent hyperglycemia, has emerged as a global epidemic, and a leading cause of morbidity and mortality worldwide. Prevention and treatment of T2DM require multiple antidiabetic agents that possess different modes of action, used in single or in combination, to control glucose homeostasis (3). Given the undesirable adverse side effects of these drugs and multifaceted pathophysiology of T2DM, therapeutic agents with distinct yet complementary actions are still urgently needed.

Plant-based diet has been increasingly recognized as a promising strategy for preventing and treating T2DM. This Research Topic, “*Flavonoids, phenolics, and saponins in the diet for prevention and management of type 2 diabetes*”, contributes to advance our understanding of plant-derived compounds, particularly flavonoids, phenolics, and saponins, as bioactive agents in glycemic control. This Research Topic explores the pharmacological effects, mechanisms of action, and antioxidant indices of plant-based diets for prevention and treatment of T2DM to bridge the gap between dietary intervention and diabetes management.

Mulberry leaf (*Folium Mori*), a botanical source rich in bioactive flavonoids, exhibits therapeutic potential in ameliorating metabolic dysfunctions associated with T2DM. The research paper entitled “Mulberry leaf ameliorate STZ induced diabetic rat by regulating hepatic glycometabolism and fatty acid β-oxidation” performed metabolomic and proteomic analysis of liver tissues from diabetic animals with or without Mulberry leaf treatment to investigate underlying mechanisms of Mulberry leaf against T2DM (Lv et al.). Their findings revealed that regulation of the glucose-lipid metabolism and amino-terminal pathways in the liver played important role in the management of T2DM by Mulberry leaf. The expression of key enzymes involved in these pathways, including ACSL5, Dlat, Pdhb, G6pc, Mdh2, were further confirmed to be regulated by Mulberry leaf.

Suaeda salsa (*Suaeda salsa* (Linn.) Pall.), rich in polyphenols, flavonoids, and other antioxidant compounds, exhibits significant nutritional potential for metabolic health. However, its therapeutic potential against T2DM remains to be elucidated. The research paper entitled “Regulation of intestinal flora by Suaeda salsa extract ameliorates hyperglycemia in a mouse model of type 2 diabetes mellitus” found that Suaeda salsa intervention significantly ameliorated hyperglycemia in T2DM mouse model. The experimental results further demonstrated that the hypoglycemic effects of Suaeda salsa were associated with the upregulated mRNA levels of Akt serine/threonine kinase (AKT-1) and glucose transporter-2 (GLUT-2). Results of microbiota analysis revealed that treatment with Suaeda salsa induced significant compositional changes in gut microbiome, particularly increasing the relative abundance of beneficial genera (Soleaferrea, Alloprevotella, Lactobacillus, and Faecalibaculum), while suppressing populations of potentially harmful bacteria (Phocaeicola and Bilophila; Yin et al.).

*Garcinia mangostana* Linn. (*Garcinia mangostana* L.) extracts have gained increasing attention as functional foods and beverages owing to their anti-inflammatory effects and weight management benefits. In a systematic review and network meta-analysis (Hypoglycemic activity of Garcinia mangostana L. extracts on diabetes rodent models: A systematic review and network meta-analysis), Chatatikun et al. demonstrated that *Garcinia mangostana* L. possesses remarkably potent hypoglycemic effects and improves lipid profiles, suggesting its promising potential as a natural therapeutic agent for diabetes management.

Emerging evidence from multicenter cohort studies elucidates the protective role of antioxidant micronutrients against diabetic retinopathy pathogenesis. Despite emerging recognition of oxidative stress in diabetic retinopathy pathogenesis, a critical knowledge gap remains regarding the relationship between composite dietary antioxidant index and diabetic retinopathy. In the review paper “The association between composite dietary antioxidant index and diabetic retinopathy in type 2 diabetic patients: evidence from the NHANES”, Liu et al. demonstrated the inverse correlation between the composite dietary antioxidant index and diabetic retinopathy risk by analyzing data from the National Health and Nutrition Examination Survey, emphasizing the potential utility of this dietary index in assessing diabetic retinopathy risk.

In summary, the published articles in this Research Topic have contributed to our understanding of the plant-derived diet as bioactive agents in glycemic control. Phytochemicals, such as flavonoids, phenolics, and saponins, are secondary metabolites produced by plants. Accumulating evidence have evinced that phytochemicals enhance the therapeutic efficacy and ameliorate side effects of drugs through complex interaction of targets. Many phytochemicals from plant-based foods and beverages, especially traditional herbal medicine, that exert less toxic and multitargeted properties have proven to be effective remedies for T2DM. However, the specific *in vivo* targets and mechanistic pathways of phytochemicals against T2DM have barely been elucidated. Future research should be devoted to the development of high-throughput platforms for screening functional compounds in diet and integrated strategy to decode the underlying therapeutic mechanisms.
